# Human Induced Pluripotent Stem Cell-Derived Cardiomyocytes Afford New Opportunities in Inherited Cardiovascular Disease Modeling

**DOI:** 10.1155/2016/3582380

**Published:** 2016-03-27

**Authors:** Daniel R. Bayzigitov, Sergey P. Medvedev, Elena V. Dementyeva, Sevda A. Bayramova, Evgeny A. Pokushalov, Alexander M. Karaskov, Suren M. Zakian

**Affiliations:** ^1^Federal Research Center, Institute of Cytology and Genetics, Siberian Branch of the Russian Academy of Sciences, Academy Lavrentyev Avenue 10, Novosibirsk 630090, Russia; ^2^Institute of Chemical Biology and Fundamental Medicine, Siberian Branch of the Russian Academy of Sciences, Academy Lavrentyev Avenue 8, Novosibirsk 630090, Russia; ^3^State Research Institute of Circulation Pathology, Rechkunovskaya Street 15, Novosibirsk 630055, Russia; ^4^Novosibirsk State University, Pirogova Street 2, Novosibirsk 630090, Russia

## Abstract

Fundamental studies of molecular and cellular mechanisms of cardiovascular disease pathogenesis are required to create more effective and safer methods of their therapy. The studies can be carried out only when model systems that fully recapitulate pathological phenotype seen in patients are used. Application of laboratory animals for cardiovascular disease modeling is limited because of physiological differences with humans. Since discovery of induced pluripotency generating induced pluripotent stem cells has become a breakthrough technology in human disease modeling. In this review, we discuss a progress that has been made in modeling inherited arrhythmias and cardiomyopathies, studying molecular mechanisms of the diseases, and searching for and testing drug compounds using patient-specific induced pluripotent stem cell-derived cardiomyocytes.

## 1. Introduction

**Figure 1:**
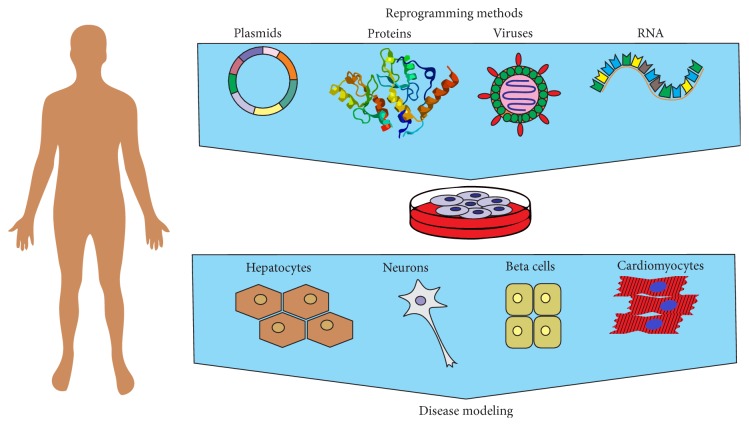
Induced pluripotent stem cell-based approach for human disease modeling. hiPSCs can be generated from human somatic cells using viruses, plasmids, modified RNA, and recombinant proteins. iPSCs are differentiated into various cell types for disease modeling, drug screening, and cell therapy.

Cardiovascular diseases (CVDs) include a wide range of diseases which greatly differ in their manifestations and underlying causes. Among CVDs there are acute conditions such as myocardial infarction, congenital heart diseases, and inherited diseases induced by genetic mutations. To search for new drugs and approaches to CVD treatment, the modern experimental medicine applies several types of model systems. First of all, these are animal models, mainly rodents: laboratory mice and rats. To date rodents are actively used to create models of acute myocardial infarction, different types of arrhythmia, and vessel diseases. Besides, there are lines of laboratory animals that carry mutations causing inherited CVDs [1, 2]. Despite the great amount of data on CVD pathogenesis and ways of their treatment obtained using laboratory animals, these model systems have some restrictions that are due to differences in the cardiovascular physiology (heart rate, peculiarities of the repolarization phase of action potential, etc.) between animals and humans (Table 1). This is particularly important in modeling of diseases caused by malfunctioning of potassium channels, as different types of potassium channels play a key role in cardiomyocyte repolarization in different species [1]. This problem can be solved by using human cardiomyocytes. However, myocardial tissue biopsy is an invasive procedure and requires a special surgical interference (e.g., abdominal operation), which cannot be carried out for the whole CVD range. In addition, the biopsy size is very small and differentiated cardiac cells have low survival and proliferation potentials. The obstacles can be overcome by techniques of generation and directed differentiation of human pluripotent cells. In 2006, Takahashi and Yamanaka reported a pioneering work on reprogramming of adult somatic cells into induced pluripotent stem cells (iPSCs) by overexpression of four transcription factors—Oct3/4, Sox2, Klf4, and c-Myc [3]. These factors are able to turn back terminally differentiated cells to a pluripotent state. iPSCs have been generated from various donor cell types—keratinocytes [4, 5], neuronal cells [6, 7], T-lymphocytes [8, 9], and adipose stromal cells [10]. Like human embryonic stem cells (hESCs), iPSCs can be differentiated into any cell type of human organism (Figure 1). Since then, iPSCs have developed into impetuous, spectacular, and groundbreaking research field promising a great and powerful approach for personalized medicine in the future. However, large-scale and safe application of iPSCs and their derivatives in substitutive cell therapy, including cardiovascular disease treatment, still requires experiments on animal models. At the same time, creation of cell models of inherited cardiovascular diseases using patient-specific iPSCs-derived cardiomyocytes is being developed.

**Figure 2:**
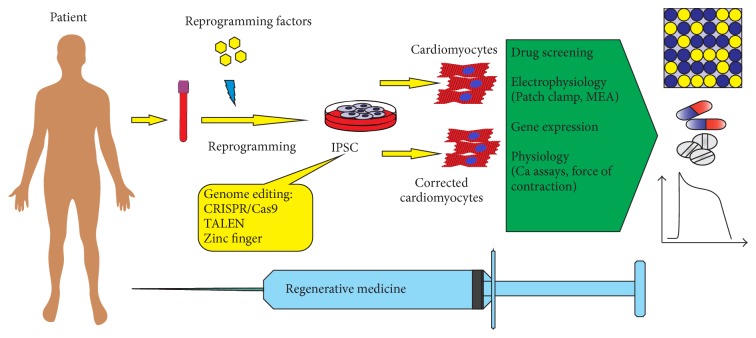
Genome editing in cardiovascular disease modeling with induced pluripotent stem cells. Patient-specific induced pluripotent stem cells are corrected using genome editing tools to create a panel of isogenic iPSC lines that are differentiated into cardiomyocytes for disease modeling, drug discovery and screening, and cell therapy.

One of the advantages of the iPSC-based approach is that iPSCs can be generated in any period of patient's life from any type of differentiated cells. Cardiomyocytes obtained during iPSC differentiation are very similar to native cardiomyocytes in morphology, gene expression pattern, electrophysiological rates, and sensitivity to chemical substances. Reliable and efficient methods of iPSC differentiation into various types of cardiomyocytes (atrial, ventricular, and nodal cardiomyocytes) are being currently established. Generating patient-specific iPSCs with subsequent differentiation into cardiomyocytes was already applied to model such CVDs as long QT syndrome, arrhythmogenic cardiomyopathy/right ventricular dysplasia, heart failure, hereditary supravalvular stenosis, catecholaminergic polymorphic ventricular tachycardia, and others. The cell models provide a good opportunity to study CVD pathogenesis at the cellular and molecular levels, search for new drugs, make toxicological researches, and examine possibilities of CVD cell and gene therapy (see Table 6) (Figure 2).

In this review, the problems concerning search for and testing of medical drugs using disease cell models are discussed. Besides, patient-specific iPSC-derived cardiomyocyte application for* in vitro* reproduction of CVD pathologic phenotype, studying CVD molecular mechanisms, and search for new therapy methods is considered.

## 2. Cardiotoxicity of Drugs

The Caucasian population is the most studied population, although risk alleles and their frequencies may vary between populations. There are approximately 150 FDA-approved drugs containing pharmacogenomic information in their labeling, describing polymorphic drug targets, genotype-specific dosing, risk of adverse dosing, or clinical response variability. However, individual genetic and epigenetic variations are usually not taken into consideration, making the drugs less effective, ineffective, or even harmful for some patients. The personalized medicine may help physicians to treat patients effectively and without risk of adverse drug reactions. The latter is one of the leading causes of hospitalization in the USA and accounts for more than 110000 deaths annually and more than 700000 serious outcomes (e.g., death, hospitalization, life-threatening disability, and congenital anomaly) [11]. According to FAERS (FDA Adverse Events Reporting System), the number of adverse drug reactions is steadily growing, which may be potentially prevented by an individualized approach to treatment. The median cost of drug developing from discovery to shelf in pharmacy is 1–5 billion US dollars. Nevertheless, while pharmaceutical companies spend money to develop new drugs that pass preclinical and clinical studies, the drugs may be further removed from market. Cardiovascular events or disorders were the main reasons for drug withdrawal in the last 10 years. About thirty drugs belonging to various drug categories (histamine antagonists, antipsoriatic agent, peripheral vasodilator, anorectic and hypolipidaemic agent, sympathomimetics, cough suppressant, antiobesity agents, anthelmintic, etc.) have been withdrawn from market because of unexpected side effects on cardiovascular system (see Table 2). Side effects can damage structure and survival of cardiomyocytes and promote myocardial infarction and stroke as is the case with the anti-inflammatory drug, Vioxx, and many anticancer drugs, such as doxorubicin [12, 13].

Heart rate and QT duration (prolongation or shortening) can also be affected, which can lead to polymorphic ventricular tachyarrhythmia, seizures, and even sudden death. Indeed, in 2010 this was the reason for the US FDA's request for withdrawal of propoxyphene, an opioid analgesic marketed by Xanodyne Pharmaceuticals, and sibutramine, an appetite depressant marketed by Abbott Laboratories. The serotonin agonist, cisapride, had caused about a hundred deaths before its use was ceased. Unexpected side effects are damaging both for companies that spend much money on drug development and promotion and for patients taking the medication.

As discussed earlier, there are significant differences in gene expression pattern and physiology of heart between species, which can hamper efficient extrapolating toxicology studies from laboratory animals to humans. According to the MHRA report in 2006, concordance between toxicities in humans and laboratory animals was concluded to be 71% when using both rodents (mice and rats) and nonrodents (dogs and monkeys). When using rodents only, the concordance was just 43% [14]. Remarkably, humans are more sensitive to drugs than rats, dogs, and mice. The latter tolerates 6–400-fold higher concentration of various antineoplastic agents compared to humans (e.g., Amethopterin, Nitromin, Cytoxan, ThioTEPA, Myleran, Pactamycin, Carboplatin, Amsacrine, Thalicarpine, Chlorozotocin, and Fludarabine) [15]. Conversely, potentially valuable drugs may be removed from the pipeline because of toxicity in animals whereas they might be completely nontoxic in humans.

To address this, the FDA proposed a new paradigm labeled the “Comprehensive* In Vitro* Proarrhythmia Assay” to assess the cardiac safety of new or existing drugs in phase 1 studies. Essential to the new paradigm is a focus on understanding mechanisms by in silico reconstruction of human cellular ventricular electrophysiology and confirmation of the electrophysiological effects on human iPSC-derived cardiomyocyte assays [16].

The reason why drugs with lethal side effects are not removed from the pipeline before they reach the clinic is the use of inadequate drug screening and safety assessment platforms. Drugs are tested for ion channel targets on immortalized cell lines (e.g., Chinese hamster ovary (CHO) or human embryonic kidney (HEK) cells) engineered to overexpress appropriate ion channels [17]. However, these cell lines do not reproduce exactly ion channel functioning under normal and pathological conditions (e.g., in case of long QT syndrome). Therefore, human cardiomyocytes are an ideal object to study CVDs. In turn, pluripotent cells, in particular iPSCs, can be an unlimited source of human cardiomyocytes. The examples of iPSC use in inherited CVD modeling are given below.

## 3. Long QT Syndrome

Long QT (LQT) syndrome is a cardiovascular disease which is diagnosed by QT interval prolongation on the ECG. QT duration depends on sex, age, and heart rate. Therefore, corrected QT interval is used. It is calculated taking into account both QT duration and heart rate. The QT interval is thought to be prolonged when duration of the corrected QT interval is more than 460 ms. According to different estimates, the prevalence of this disease is 1 : 2000–1 : 3000. QT interval prolongation is caused by extension of repolarization during action potential in ventricular cardiomyocytes. This increases the risk of early afterdepolarization, which, in turn, can cause polymorphic ventricular tachycardia. The most frequent ventricular tachycardia in LQT syndrome is torsade de pointes which leads to syncope and may transform into ventricular fibrillation causing the cardiac arrest and sudden death [18].

There are two forms of LQT syndrome, which are acquired and congenital [19]. Acquired form can be caused by a wide range of reasons like other CVDs, disorders of electrolyte exchange, central nervous system diseases, endocrine abnormalities, stress, abstinence from food, diets, poisoning by chemical compounds, and side effects of some drugs. The list of drugs which can prolong the QT interval is being expanded. It includes antiarrhythmic, antihistamine, antibiotic, anaesthetic, and antidepressant drugs and many others. Congenital form is due to mutations in genes encoding proteins involved in potassium, sodium, and calcium channel structure and functioning in cardiomyocytes. The majority of the mutations is autosomal-dominant. To date 13 genes have been identified, mutations in which induce LQT syndrome. According to the genes, there are 13 types of congenital syndrome (LQT1–LQT13) (see Table 3). It is worth mentioning that the first three types are most common; they account for more than 90% of all affected by congenital LQTS [19].

Nowadays, beta blockers as well as implantable cardioverter-defibrillators or cardiostimulators are used to treat LQT syndrome. However their efficiency is insufficient which requires developing new approaches to LQTs treatment. The problem is that the disease is very heterogeneous. There are hundreds of LQT syndrome causing mutations that are located in different parts of the genes and lead to different severity of disease manifestations. Moreover, carriers of the same mutation (even within one family) may demonstrate different disease severity—from early onset of cardiac events to absence of any symptoms. The factors defining LQT syndrome severity need to be clarified. Nevertheless, sex and age (level of sex hormones), other diseases, and administration of some drugs are believed to influence the QT interval duration [20, 21]. Additionally, amount of data on correlations between some single nucleotide polymorphisms (SNPs), QT duration, and disease severity is currently increasing [20, 22, 23]. This heterogeneity requires personalized approaches to LQT syndrome therapy, which can not be provided by existing animal models and heterogeneous systems. iPSC-based technology opened new perspectives in creating more advanced personalized models of LQT syndrome. Several groups have already generated patient-specific iPSC lines carrying mutations causing LQT syndrome types 1, 2, 3, and 8 (see Table 3), that is, for four types of congenital syndrome out of thirteen. The patient-specific iPSC lines were differentiated into cardiomyocytes that showed increase in action potential duration and disturbances in functioning of ion channels involved in appropriate type of LQT syndrome.

iPSC-derived cardiomyocytes to model LQT syndrome were firstly obtained in 2010. Two patients, representatives of one family, carrying the missense mutation c.569G>A (p.R190Q) in* KCNQ1* encoding alpha-subunit of slow delayed rectifier potassium current (*I*_Ks_) channels were used. The R190Q mutation was found to disrupt KCNQ1 trafficking to cell membrane, which resulted in reduced number of *I*_Ks_ channels in the patients. As a result, *I*_Ks_ decreased by 70–80% in the patient-specific cardiomyocytes as compared to control ones. Patient-specific cardiomyocytes were also prone to catecholamine-induced tachycardia that could be managed by beta blockers [24]. In another study, patient-specific iPSCs having the 1893delC mutation in* KCNQ1* were generated [25]. The electrophysiological analysis of contracting areas of embryoid bodies (EBs) showed significant increase in the field potential duration. The blocker of fast delayed rectifier potassium current (*I*_Kr_) channels, E4031, greatly increased the field potential duration in both control and patient-specific EBs. However, E4031 induced arrhythmia only in patient-specific EBs. At the same time, *I*_Ks_ channel blocker, chromanol 293B, increased the field potential duration in control EBs, but not in patient-specific EBs. This fact suggests that LQT syndrome was due to disorders in *I*_Ks_ channel functioning. Studying individual patient's cardiomyocytes by patch-clamp and immunocytochemistry showed that the 1893delC mutation also interfered with mutant protein trafficking from cytoplasm to cell membrane [25]. These studies confirm that cardiomyocytes obtained in the course of patient-specific iPSC differentiation adequately reproduce the main features of the pathological phenotype of LQT syndrome type 1.

iPSC-based models were also created for LQT syndrome type 2. Patient-specific iPSCs carrying different mutations in* KCNH2* that encodes alpha-subunit of *I*_Kr_ channels have been generated. Electrophysiological studies of the iPSC-derived cardiomyocytes revealed a number of pathological manifestations. For example, cardiomyocytes obtained from the patient having the missense A614V mutation in* KCNH2* showed a decrease in *I*_Kr_ (by about 60%) and increase in duration of action and field potentials as compared to control cells [26]. In addition, the cardiomyocytes demonstrated signs of early afterdepolarization (about 66% of cells) and premature contractions (about 36% of cells). The interesting fact is that action potential prolongation and early afterdepolarization were greatly inhibited while using nifedipine, a blocker of calcium channels, and pinacidil that stimulates opening of ATP-dependent potassium channels.

Patient-specific iPSC-derived cardiomyocytes can be used for search for and study of new drug combinations as was successfully demonstrated in the study by Matsa et al. [27]. iPSCs were obtained from a fifteen-year-old female that showed symptoms of LQT syndrome and carried the missense c.1681G>A (p.A561T) mutation in* KCNH2* and her mother who was an asymptomatic carrier of the mutation. Cardiomyocytes obtained from both daughter's and mother's iPSCs showed prolonged field/action potential duration as compared to control cells. However, the daughter's cardiomyocytes had a more pronounced elongation of the action potential. Adding of isoprenaline shortened the action potential duration but caused early afterdepolarization in a great number of cardiomyocytes. Effect of isoprenaline was reversed by beta blockers (nadolol and propranolol). Use of potassium channel activators, nicorandil and/or PD-118057, also led to decrease in the action potential duration; however, no arrhythmogeniс early afterdepolarization was observed [27]. Another study showed that roscovitine was able to deactivate L-type calcium current (*I*_Ca,L_) channels and restore normal calcium currents and electrophysiological properties of cardiomyocytes derived from iPSCs of Timothy syndrome patients [5]. This syndrome is associated with autosomal-dominant mutations in* CACNA1C* encoding alpha-subunit of *I*_Ca,L_ channels and is accompanied not only by QT prolongation and arrhythmias (LQT syndrome type 8) but also by a wide range of disorders, congenital heart disorders and syndactyly and autism and backwardness and high risk of sudden death at an early age [28].

Thus, iPSC-derived cardiomyocytes of particular patients can be used for selection of individual drug combinations, which may help to avoid complications during drug therapy. This approach is strongly supported by the fact that patients' iPSC-derived cardiomyocytes are able to adequately reproduce individual peculiarities of disease manifestation. For example, cardiomyocytes of a patient having the R176W mutation in* KCNH2* demonstrate a significant increase in action and field potentials but do not show any signs of early afterdepolarization, which agrees with the absence of arrhythmic events in this patient [29]. The relationships between genotype and clinical symptoms seen in patients were fully reproduced in iPSC-based models for LQT syndrome types 3 and 8 [5, 30, 31].

Cardiomyocytes obtained from iPSCs of LQT syndrome patients can be also a tool for development and testing new therapy methods. In one study, allele-specific RNA interference was used to correct a mutant phenotype in patient-specific cardiomyocytes [32]. Using short interfering RNA, the authors managed to decrease the RNA level of a mutant* KCNH2* (*hERG*) allele (c.G1681A) by 61.8%, thereby increasing probability of formation of functional hERG tetramer by 4.5 times. It was enough to normalize the action potential duration, to restore *I*_Kr_, and to reduce the frequency of spontaneous and induced arrhythmias (early afterdepolarization) [32]. This fact suggests that the allele-specific RNA interference can be an efficient method to correct LQT syndrome type 2 and other diseases with autosomal-dominant inheritance. However, to apply this method in clinic, a number of difficult issues need to be solved. One of them is targeted siRNA delivery in specific cell types of affected organ, for example, in cardiomyocytes. Experiments on mouse LQT syndrome models may be required to find a solution of this issue [33, 34].

In addition, cardiomyocytes obtained from patient-specific iPSCs can be used to test newly detected mutations, to clarify relationships between the mutations and disease phenotype, and to study molecular mechanisms of disease pathogenesis. For example, Bellin et al. obtained iPSCs of a female LQT syndrome patient who was asymptomatic and diagnosed only based on electrocardiogram results [35]. The genetic screening showed that the patient had the c.A2987T (p.N996I) mutation in* KCNH2*. In order to verify the role of this mutation in LQT syndrome development, two pairs of isogenic pluripotent cell lines that had the same genetic background and differed only by one point mutation were used. One pair was two patient-specific iPSC lines. One carried the N996I mutation whereas in the other the mutation was corrected by homologous recombination. The second pair was two ESC lines that differed in presence/absence of the N996I mutation. Electrophysiological analysis of the iPSC- and ESC-derived cardiomyocytes showed that only the presence of the N996I mutation increased the action potential duration and damaged *I*_Kr_. Correction of the mutation improved these parameters. Further studies demonstrated that this mutation disrupted hERG trafficking to cell membrane, which seemed to underlie pathology development [35].

Thus, iPSC-derived cardiomyocytes of LQT syndrome patients are being used to study molecular mechanisms of disease development, to examine new disease causing mutations, to search for new therapy methods, and to test existing drugs for QT interval prolongation and proarrhythmic activity.

## 4. Catecholaminergic Polymorphic Ventricular Tachycardia

Catecholaminergic polymorphic ventricular tachycardia (CPVT) is a severe inherited cardiovascular disease. Its prevalence is about 1 : 10000. CPVT is defined by absence of structural heart damage and severe tachycardia induced by physical or emotional stress [36]. CPVT causes syncope and is a frequent reason of sudden death among young people [37]. About 30% of CPVT patients show the symptoms under the age of 10, and the death rate under the age of 35 is 30–35%. The only methods of CPVT therapy are beta blockers and automatic cardioverter-defibrillator implantation [38]. Mutations in two genes are known to cause CPVT. One of the genes* (RyR2)* encodes a ryanodine receptor. This protein is involved in calcium ions release from sarcoplasmic reticulum and plays a key role in cardiomyocyte contractions. There are over 150 mutations in* RyR2* which have autosomal-dominant inheritance, cause CPVT type I, and are responsible for up to 55% of CPVT cases. Mutations in another gene* (CASQ2)*, which encodes the calcium binding protein (calsequestrin), are much rarer and cause about 3–5% of CPVT cases. 15 mutations were identified in* CASQ2*. The mutations have autosomal-recessive inheritance and cause CPVT type II [39].

To model CPVT* in vivo*, mice that had mutations in* RyR2* or were* CASQ2* knockouts were used. To study the disease* in vitro*, myocytes were transduced with recombinant adenoviruses expressing mutant CASQ2 forms. Both model types were found to reproduce the CPVT clinical pattern including delayed afterdepolarization in response to adrenergic stimulation [40–44]. However, the method of inducing the pluripotent state in somatic cells has opened new perspectives in studying mechanisms of this severe disease and searching for new ways of its treatment.

A research group generated iPSCs of patients from a Bedouin tribe living in the northern part of Israel and having rare homozygous missense p.D307H mutation in* CASQ2* [39]. Beta-adrenergic agonist (isoproterenol) stimulation of the patient-specific iPSC-derived cardiomyocytes caused delayed afterdepolarization, arrhythmogenic oscillating prepotentials, and postcontractions. The effects were not detected in control cardiomyocytes [39]. Analysis by electronic microscopy showed that the patient-specific cardiomyocytes had immature phenotype, less organized myofibrils, enlarged cisterns of sarcoplasmic reticulum, and reduced number of caveolae [39]. The patients' cardiomyocytes were also defined by decreased contraction frequency. This agreed with clinical data, according to which CPVT type II patients had bradycardia at rest.

Using patient-specific iPSCs, CPVT type I models were also obtained [45–47]. Fatima et al. generated iPSCs of a 46-year-old woman having the dominant missense p.F2483I mutation in* RyR2* which was within FKBP12.6-binding domain of ryanodine receptor [45]. Unlike control cardiomyocytes, isoproterenol treatment of the patient-specific cardiomyocytes resulted in a negative chronotropic effect, delayed afterdepolarization, and arrhythmia. Visualization of calcium ion currents in the patient's cardiomyocytes showed a high amplitude and duration of calcium ion release, even without adrenergic stimulation. Similar results were obtained upon studying cardiomyocytes of a 25-year-old male patient having the missense p.P2328S mutation in* RyR2* [47]. As was shown by patch-clamp, the patient-specific cardiomyocytes had the signs of delayed afterdepolarization in case of spontaneous contractions and in presence of adrenaline as well as early afterdepolarization in case of spontaneous contractions. Besides, the patient's cardiomyocytes had a lower content of calcium ions in the sarcoplasmic reticulum, which suggested its possible abnormal leakage [47].

iPSC-based model of CPVT type I was also created for a female patient carrying the p.S406L mutation in the RyR2 N-terminal domain [46]. Cardiomyocytes of a healthy donor and the patient showed that they had similar levels of calcium ions in systole and diastole and equal content of calcium ions in the sarcoplasmic reticulum. However, during isoproterenol stimulation the level of calcium ions in diastole sharply increased in the patient's cells as compared to control ones, while the level of calcium ions in systole did not change. The calcium ion content in sarcoplasmic reticulum did not increase in the patient's cardiomyocytes in response to isoproterenol. Under the intact condition in the patient-specific cardiomyocytes, abnormal sparks of calcium ion release and more extended plateau phase and decay phase were observed. In response to isoproterenol the frequency of such sparks increased and they acquired longer decay phases. The use of dantrolen (the drug effectively used for treatment of the malignant hyperthermia) was shown to normalize parameters of calcium ion release sparks and prevent cells from arrhythmia [46].

Thus, as in the case of LQT syndrome, patient-specific iPSC-derived cardiomyocytes adequately reproduce the disease* in vitro* and are a good tool for studying CPVT molecular mechanisms and drug searching (see Table 4).

## 5. Inherited Cardiomyopathies

Cardiomyopathies (CMPs) are a group of cardiovascular diseases that are defined by structural and functional changes of the cardiac muscle forming even in the absence of coronary artery pathologies, increased arterial pressure, or cardiac valvulopathy. There are several types of primary cardiomyopathies: arrhythmogenic right ventricular dysplasia, dilated (congestive) CMP, hypertrophic CMP, specific CMP (metabolic, inflammatory, ischemic, cirrhotic, etc.), and unclassified CMP (fibroelastosis, noncompaction cardiomyopathy (spongiform cardiomyopathy), mitochondriopathies, etc.). Primary CMPs can be caused by viruses, bacteria, autoimmune disorders, toxic action of the alcohol, and medical drugs and also by genetic mutations. In most cases, the only way of CMP therapy is cardiac transplantation. In 2012-2013, several research groups obtained cell models of inherited arrhythmogenic right ventricular dysplasia (ARVD) and dilated (DCMP) and hypertrophic (HCMP) cardiomyopathies using patient-specific iPSCs (see Table 5) [31, 48–52].

## 6. Arrhythmogenic Right Ventricular Dysplasia (ARVD)

ARVD is defined by progressive replacement of healthy tissue of cardiac muscle, mainly in the right ventricle, with fibroadipose tissue and enhanced apoptosis of cardiomyocytes. These changes lead to ventricular tachycardia and increased risk of sudden death. Despite the disease severity and high incidence, there are still few data on the mechanisms of its onset and development. This mainly is due to low availability of the material (myocardial biopsy) for research, especially at the early stages of disease development. Nearly 50% of patients suffering from ARVD have mutations in genes which encode desmosomes components—desmoplakin, plakoglobin, plakophilin-2, desmoglein-2, and desmocollin-2 [53]. Mutations in* PKP2* encoding plakophilin-2 are the most frequent. In three studies, iPSC-derived cardiomyocytes of patients with diagnosed ARVD have been obtained [48, 49, 54]. In one case, a patient was homozygous for the c.2484C>T mutation in* PKP2* [49]. This mutation interrupts transcript splicing and induces 7-nucleotide deletion in the* PKP2* exon 12. Мain pathological hallmarks of ARVD are progressive fibrofatty replacement of cardiomyocytes with increased cardiomyocyte apoptosis. However, changes in plakoglobin localization in patient iPSC-derived cardiomyocytes did not influence lipogenesis and apoptosis in mutant cardiomyocytes as compared to the control ones. The fact is that iPSC-derived cardiomyocytes were immature and corresponded to embryonic cardiomyocytes. To produce energy embryonic cardiomyocytes use glycolysis while adult cardiomyocytes do fatty acid oxidation. In order to reproduce ARVD pathogenesis* in vitro*, energy metabolism of adult cardiomyocytes was activated with insulin, dexamethasone, and 3-isobutyl-1-methylxanthine. The stimulation activated transcription of the main regulator of fatty acid oxidation, PPAR- (peroxisome proliferator-activated receptor-) alpha, but this did not induce statistically significant changes in the mutant cardiomyocytes properties. Then adipogenic differentiation in combination with rosiglitazone and indomethacin that are activators of the PPAR-gamma receptor was applied. Activation of the PPAR-gamma signaling cascade, which is abnormally active in cardiac muscle of ARVD patients, resulted in enhanced lipogenesis and apoptosis in the iPSC-derived mutant cardiomyocytes. Interestingly, insulin, dexamethasone, and 3-isobutyl-1-methylxanthine used for PPAR-alpha activation adequately reproduced hormone action in adult human body. At the same time, there are no ligands activating the PPAR-gamma and similar to rosiglitazone and indomethacin in their chemical structure. Nevertheless, it was found that rosiglitazone and indomethacin could be replaced with 13-hydroxy-octadecadienoic acid, main component of oxidized low-density lipoproteins, during PPAR-gamma activation. Application of PPAR-gamma antagonists (GW9662 and T0070907) upon reproducing the pathological condition could prevent cardiomyocyte lipogenesis and apoptosis. Finally, pathological features were shown to be inhibited by either PPAR-alpha inactivation or PPAR-gamma activation only. This means that pathological mechanisms involve activation of both PPAR-alpha and PPAR-gamma signaling cascades. The conclusion was confirmed using iPSC-derived cardiomyocytes of a patient heterozygous for the c.2013delC deletion inducing frameshift mutation and transcription termination in the* PKP2* exon 10 [49].

In the other two studies, iPSC-derived cardiomyocytes of patients carrying two different mutations in* PKP2* were generated. One mutation was the heterozygous c.972InsT/N insertion leading to frameshift mutation [48] and the other was the missense c.1841T>C (p.L614P) mutation [54]. Increased lipogenesis, damage of desmosome structure, and PPAR-gamma activation were observed in the patient-specific iPSC-derived cardiomyocytes. One of the studies also showed that the adipogenic stimulation intensified changes in the desmosomes structure and lipid accumulation. These effects could be prevented by a specific inhibitor of glycogen synthase kinase 3 beta [48].

## 7. Dilated Cardiomyopathy (DCM)

Dilated cardiomyopathy is a myocardial disease characterized by ventricular chamber enlargement and systolic dysfunction with no increase in ventricular wall thickness. DCM is one of the most common reasons of cardiac failure after coronary vessels disease and elevated blood pressure [55, 56]. About 30–35% of DCM cases are inherited. Mutations in more than 30 genes encoding proteins of cytoskeleton, sarcomere, and nuclear lamina can cause DCM [57].

iPSC-derived cardiomyocytes have been also obtained for patients with inherited DCM [51, 52]. In one study, a patient demonstrated such cardiological symptoms as palpitation and precollaptoid states as well as nonsustained ventricular tachycardias were detected by the Holter monitoring. The patient's father and brother suddenly died because of nondefined cardiac disease. Exome sequencing revealed previously unknown heterozygous missense с.940C>T (p.A285V) mutation in* DES* encoding desmin [52].* DES* mutations were previously shown to induce various forms of DCM [57–59]. iPSC-derived cardiomyocytes of the patient carrying the с.940C>T mutation had a number of structural abnormalities such as presence of diffuse desmin-containing aggregates and weak interaction of desmin with troponin T, alpha-actin, and F-actin. Scanning electron microscopy analysis showed structure damage of sarcomere Z-disks and presence of pleomorphic dense structures near Z-disks or between myofibrils. In comparison with control cardiomyocytes, the patient-specific ones demonstrated a lower contractile rate and inadequate response to adrenergic stress induced by isoproterenol [52].

In another study, genetic screening of 7 family members was performed to identify DCM causing mutation [51]. Four individuals, representatives of three generations, had the missense p.R173W mutation in* TNNT2* which encodes cardiac troponin T. iPSC-derived cardiomyocytes were generated for each mutation carrier. The mutant cardiomyocytes demonstrated changes in regulation of calcium ion currents and elevated contractility and most cells had irregular distribution of sarcomeric alpha-actin. Beta-adrenergic agonists increased intensity of the pathological changes, while beta blockers or ectopic expression of Serca2a (calcium-dependent adenosine triphosphatase of sarcoplasmic reticulum) restored the functions in the mutant cardiomyocytes [51].

DCM may also be a trait of some complex inherited syndromes. For example, DCM is a symptom of the Barth syndrome along with skeletal muscle myopathy, neutropenia, growth retardation, and 3-methylglutaric aciduria. The Barth syndrome is caused by mutations in* TAZ1* located on X-chromosome (Xq28) and encoding mitochondrial protein tafazzin. Cell models based on iPSCs of patients having* TAZ1* mutations have been successfully obtained and used to study effect of the mutations on mitochondria functioning [60].

A recent study showed that cardiomyocyte treatment with antisense oligonucleotides to Ser14450fsX4 mutation in the TTN exon 326 improves DCM phenotype at both structural and functional levels in mice and patient-derived cells. Skipping of the exon with Ser14450fsX4 mutation in patient cardiomyocytes improved myofibril assembly and stability and normalized expression of TTN regulated genes. TTN knock-in in homozygous and heterozygous mice confirmed the effect of exon skipping. This may potentially provide more effective treatment and early prevention of heart failure. However, preclinical research is needed to determine the optimal regimen of treatment with antisense oligonucleotides and to make pharmacokinetic/pharmacodynamic analysis [61].

## 8. Inherited Hypertrophic Cardiomyopathy

Inherited hypertrophic cardiomyopathy (HCM) is an autosomal-dominant disease which is characterized by structural damage of cardiomyocyte sarcomeres. HCM patients have abnormal increase in left ventricle wall thickness in the absence of enhanced haemodynamic activity and high risk of progressive cardiac failure, arrhythmia, and sudden cardiac death. HCM is one of the most common inherited cardiovascular diseases [55].

In 2013, an iPSC-based model of HCM was created [50]. iPSCs were generated from a family consisting of ten individuals. In the family, mother and four children had missense p.R663H mutation in* MYH7* encoding beta-myosin heavy chain. iPSC-derived mutant cardiomyocytes demonstrated HCM features—enlarged cell size and arrhythmia. The cardiomyocytes had irregular circulation of calcium ions and their content in the cells was highly increased, which was the main mechanism of disease pathogenesis. Furthermore, several drugs that had prevented hypertrophy and electrophysiological disorders were identified [50].

Hypertrophic cardiomyopathy is the main manifestation of a rare inherited syndrome—the LEOPARD syndrome. This name is the acronym of the words denoting its manifestations: lentigines, electrocardiographic abnormalities, ocular hypertelorism, pulmonary valve stenosis, abnormal genitalia, retardation of growth, and deafness. Nearly 90% of the LEOPARD syndrome cases and 45% of Noonan's syndrome cases are caused by missense mutations in* PTPN11* encoding the SHP2 tyrosine phosphatase. In 2010, iPSC-derived cardiomyocytes of two patients having missense p.T468M mutation in* PTPN11* were generated. The patients' cardiomyocytes had a larger size and irregular sarcomere organization and most cells had nuclear localization of NFATC4 which belongs to transcriptional factors involved in hypertrophy development [62].

## 9. Supravalvular Aortic Stenosis

Supravalvular aortic stenosis (SAS) is a serious disease accompanied by increased proliferation of vascular smooth muscle cells. This results in stenosis and blocking of ascending artery and other major arteries. Patients suffering from the disease are under high risk of sudden cardiac death. Its therapy includes surgical vessel correction, vessel prosthesis, and cardiac transplantation [63]. SAS is caused by heterozygous mutations in* ELN* or deletions in the q-arm of chromosome 7 (7q11.23, Williams-Beuren syndrome) which also involve* ELN*. As deletions in 7q11.23 usually involve up to 28 genes, Williams-Beuren syndrome patients have a more complex phenotype including craniofacial defects and neurobehavioral disorders. Although the Williams-Beuren syndrome is a rare disease with prevalence of 1 : 10000, it is one of the most common vessel diseases having proven inherited nature [63, 64].

*ELN* encodes the monomeric precursor protein (tropoelastin) which is secreted by arterial smooth muscle cells. Tropoelastin polymerizes and forms elastin which is the main component of extracellular matrix of smooth muscle cells defining vessel elasticity and resistance to constant dynamic action. At present, there are SAS models obtained using laboratory animals [65, 66]. However, application of the models is limited because of functional differences between animal and human smooth muscle cells. Studying SAS is also complicated by insufficient biopsy material of patients and low viability of smooth muscle cells in culture [67, 68]. Thus, using patient-specific iPSCs as well as their directed differentiation into smooth muscle cells seems to be a very prospective tool for generating SAS models.

Such iPSCs have been obtained from a patient having 4-nucleotide insertion in* ELN* causing a frameshift mutation and premature transcription termination in exon 10 and from Williams-Beuren syndrome patients [4, 69]. As compared to the control cells, the patient-specific iPSC-derived smooth muscle cells were found to have abnormal organization of alpha-actin filaments, high rate of proliferation and migration, low sensitivity to vasoactive drugs (carbachol and endothelin-1), and decreased capacity of vessel-like structure formation [4, 69]. Application of recombinant elastin and small GTPase RhoA activation by tropomyosin allowed correcting the process of actin filament formation [69]. Furthermore, a specific kinase inhibitor ERK1/2 or rapamycin (mTOR signaling cascade inhibitor) significantly reduced abnormal rate of mutant cells proliferation [4, 69].

## 10. Problems and Prospects of Creating and Application of Inherited Cardiovascular Disease Cell Models

Protocols allowing highly efficient iPSC differentiation into cardiomyocytes have been developed. In some protocols, treatment of embryoid bodies (EBs) with different combinations of growth factors was used [70–72]. Later, protocols based on monolayer differentiation and TGF-beta subfamily receptor stimulation with activin A and bone morphogenetic protein 4 (BMP4) were developed [73, 74]. Another approach to cardiac monolayer differentiation is WNT signaling pathway activation with GSK3 protein kinase inhibitor, CHIR99021, followed by WNT repression with IWP2 [75]. An optimized inexpensive and simple cardiac differentiation protocol using metabolic selection for cardiomyocyte enrichment was also developed [76].

Although protocols of directed cardiac differentiation are being improved, most iPSC-CMs are known to have immature phenotype. Although iPSC-CMs express relevant ion channel genes (SCN5A, KCNJ2, CACNA1C, KCNQ1, and KCNH2), structural genes (MYH6, MYLPF, MYBPC3, DES, TNNT2, and TNNI3), and transcription factors (NKX2.5, GATA4, and GATA6) [77], they differ from adult ventricular cardiomyocytes in a number of properties. iPSC-CMs have smaller cell size, exhibit reduced inward rectifier K currents and the presence of prominent pacemaker currents, and manifest spontaneous membrane depolarizations. In addition, lack of t-tubules and disorganized sarcomeres are observed in iPSC-CMs. The relative immaturity of iPSC-CMs limits their usage in disease modeling, drug screening, and regenerative medicine [78]. Attempts to circumvent this limitation have shown that long-term cultivation of iPSС-CMs improves sarcomere organization [79]. In addition, external signals, such as electrical stimulation and mechanical cyclic stretching, were reported to promote functional iPSC-CM maturation [80, 81].

To date a great number of experimental evidences confirm that cell models can reproduce* in vitro* various aspects of disease pathologic phenotype. In addition, there are successful attempts to create systems to test drugs for their ability to treat disease symptoms at the cell level and their potential cardiotoxicity [82]. However, the number of iPSC lines with a certain genotype obtained for each disease is limited. Therefore, researches have to use cells carrying a limited spectrum of mutations whereas the real number of disease causing mutations can be tens or even hundreds of times higher. For example, more than 1400 mutations in more than 20 genes are known for inherited hypertrophic cardiomyopathy [83]. Such genetic diversity can cause variability in disease manifestations, which may require different approaches to disease therapy [84, 85]. This problem can be solved by creating biobanks of patient-specific iPSCs which would cover a wide range of genotypes including the rare ones (reviewed in [86, 87]). In the biobanks patient-specific iPSC lines are fully characterized, stored under common conditions, and licensed for use in research. The cell lines from the biobanks can be available to a wide range of researchers from academic institutions and pharmaceutical companies.

Another problem is selection of control cells for modeling and studying inherited cardiovascular diseases. Control cells are usually obtained from healthy people so they have another genetic background (SNPs set). Moreover, pluripotent cell lines can differ from each other in their differentiation potentials, proliferation rates, and other features, even if they were generated by one method and in one laboratory. The peculiarities may have effect on accuracy of research results. The problem can be solved by using new methods of genome engineering such as homologous recombination mediated by TALENs (Transcription Activator-Like Effector Nucleases) and CRISPR (Clustered Regularly Interspaced Short Palindromic Repeats)/Cas9. The method allows correcting existing mutations in genomes of pluripotent cells and creating so-called isogenic pluripotent cell lines that differ from each other in only one mutation. TALENs and CRISPR/Cas9 can also be used to introduce new mutations in pluripotent cell genomes. This is especially important in the case of rare mutations because the problem of availability of patients carrying the rare mutations is still acute. Genetic constructions that express dominant-negative forms of proteins and are placed in safe harbor loci, such as* AAVS1*, can be applied as well [35, 88]. Besides, the CRISPR/Cas9 system allows studying gene functions and their interaction during formation of a complex pathological phenotype. Moreover, analysis of gene influence on manifestation of phenotypic features can be carried out on genome-wide scale [89, 90]. This is essential for inherited diseases with incomplete penetrance, and this makes it possible to detect modifying genes. Knowledge of existing gene networks, modifying genes, and individual SNPs can give additional possibilities for genetic testing of patients, prediction of disease state pattern, and personalized development of optimal treatment strategy.

Thus, creation of more modern collection, storage, and distribution systems of patient-specific cell lines, as well as new genome editing technologies, can significantly promote application of cardiovascular disease cell models in translational biomedical testing.

## 11. Conclusion

Search for safer and more effective methods of cardiovascular disease therapy is one of the most important trends in modern pharmacology. Among all cardiovascular diseases, inherited ones are of special interest. Most of them have Mendel inheritance pattern. Several hundreds of disease causing mutations have been described. However, disease pathogenesis at the molecular and cellular levels is still poorly understood in most cases. Studying patient-specific iPSC-derived cardiomyocytes has shown that they are able to reproduce most peculiarities of pathological phenotype, such as electrophysiological abnormalities, sensitivity to some drugs, and other factors. iPSC-derived cardiomyocytes can be also used as platforms for drug testing that may become a basis for large-scale screening of small-molecular compound libraries. Additionally, the models can be successfully applied to study new methods of genome engineering, for example, TALENs and CRISPR/Cas9. To date obtaining a collection of iPSC lines that correspond to maximum genetic diversity (mutation variants and disease modifying SNPs), creating biobanks of cell models, improving methods of directed iPSC differentiation into cardiomyocytes, and scaling of the technology are current tasks of regenerative medicine.

## Figures and Tables

**Table 1:** Electrophysiological characteristics of heart that differ in humans and laboratory animals (modified from [91]).

	Mouse	Rat	Guinea pig	Rabbit	Dog	Human
Heart rate (bpm)	500	300	230	200	70	75
Coronary collaterals	Variable	Low	High	None	Middle	Low
Ventricular AP duration (ms)	25–40	50	140	120–140	250	250
Primary repolarizing current	*I*_Kto_	*I*_Kto_	*I*_Kr,s_	*I*_Kr,s_	*I*_Kr,s_	*I*_Kr,s_
Q wave in ECG	No	No	Yes	Yes	Yes	Yes
ST segment in ECG	No	No	Yes	Yes	Yes	Yes

**Table 2:** List of drugs withdrawn from the market for safety reasons because of severe cardiovascular effects.

Drug name	Drug class or use	Year of withdrawal	Adverse reaction or safety concern
Astemizole	Histamine antagonists	1999	Fatal arrhythmia
Azaribine	Antipsoriasis	1976	Thromboembolism
Buflomedil	Peripheral vasodilator	2011	Neurological and cardiac disorders
Benfluorex	Anorectic and hypolipidemic	2009	Risk of heart valve disease
Chlorphentermine	Sympathomimetics	1969	Cardiovascular toxicity
Cisapride monohydrate	Serotonin receptor agonists	2000	Fatal arrhythmia
Cloforex	Sympathomimetic	1969	Cardiovascular toxicity
Clobutinol	Cough suppressant	2007	QT prolongation
Dexfenfluramine	Serotonin uptake inhibitors, antiobesity agents	1997	Cardiac valvular disease
Dithiazanine iodide	Anthelmintic	1964	Cardiovascular and metabolic reaction
Dofetilide	Antiarrhythmia agents, potassium channel blockers	2004	Prolonged QT
Encainide HCl	Antiarrhythmic, sodium channel blockers	1991	Cardiotoxicity, ventricular arrhythmias
Fenfluramine	Sympathomimetic, serotonin uptake inhibitors	1997	Cardiac valvular disease
Grepafloxacin	Antimicrobial	1999	QT prolongation
Levomethadyl acetate HCl	Analgesics, opioid	2003	Cardiac arrhythmias and cardiac arrest
Mibefradil dihydrochloride	Calcium channel blockers	1998	Fatal arrhythmia
Orciprenaline	Sympathomimetic, bronchodilator, tocolytic	2010	Cardiac side effects, mainly palpitations and tachycardia
Pergolide mesylate	Dopamine agonists, antidyskinetics	2007	Risk for heart valve damage
Prenylamine	Vasodilator, calcium channel blockers	1988	Polymorphic ventricular tachycardia and death
Propoxyphene	Analgesics, opioid	2010	Increased risk of heart attacks and stroke
Rofecoxib	COX-2 selective NSAID	2004	Risk for heart attack and stroke
Rosiglitazone	Antidiabetic treatment	2011	Risk of heart failure
Sertindole	Antipsychotic	1998	Arrhythmias and sudden cardiac death
Sibutramine	Appetite depressants	2010	Cardiovascular disorders
Sparfloxacin	Fluoroquinolone antibiotic	2001	QT prolongation
Tegaserod maleate	Serotonin receptor agonists	2007	Risk for heart attack and stroke and Unstable angina
Terfenadine	Histamine antagonists	1998	Cardiovascular toxicity, prolonged QT interval
Terodiline	Antispasmodic	1991	Ventricular tachycardia and arrhythmia
Thioridazine	Antipsychotic, dopamine antagonists	2005	cardiac disorders
Valdecoxib	Nonsteroidal anti-inflammatory	2005	Risk for heart attack and stroke

**Table 3:** Human IPSC-derived patient-specific LQT syndrome cell models.

Syndrome type	Gene	Protein	Mutation	Donor cell types	Reprogramming method	References
LQT1	*KCNQ1*	Potassium voltage-gated channel subfamily KQT member 1	p.R190Q	Fibroblasts	RV^a^, OSKM^b^	[24]
			1893delC (P631fs/33)	Fibroblasts	LV^c^, OSKM	[25]

LQT2	*KCNH2*	Potassium voltage-gated channel subfamily H member 2	p.A614V	Fibroblasts	RV, OSK^b^	[26]
			p.A561T	Fibroblasts	LV, ONSL^b^	[27]
			p.R176W	Fibroblasts	RV, OSKM	[29]
			p.G603D	T-lymphocytes	SV^d^, OSKM	[92]
			p.N996I	Fibroblasts	RV, OSKM	[35]

LQT3	*SCN5A*	Sodium channel protein type 5 subunit alpha	p.F1473C	Fibroblasts	RV, OSKM	[30]
			p.V1763M	Fibroblasts	mRNA, OSKM	[31]

LQT4	*ANK2*	Ankyrin-2				

LQT5	*KCNE1*	Potassium voltage-gated channel subfamily E member 1				

LQT6	*KCNE2*	Potassium voltage-gated channel subfamily E member 2				

LQT7	*KCNJ2*	Inward rectifier potassium channel 2				

LQT8	*CACNA1*	Voltage-dependent P-type/Q-type calcium channel subunit alpha-1A	p.G406R	Fibroblasts	RV, OSKM	[5]

LQT9	*CAV3*	Caveolin-3				

LQT10	*SCN4B*	Sodium channel subunit beta-4 precursor				

LQT11	*AKAP9*	A-kinase anchor protein 9				

LQT12	*SNTA1*	Alpha-1-syntrophin				

LQT13	*KCNJ5*	G protein-activated inward rectifier potassium channel 4				

RV^a^: retroviruses, LV^c^: lentiviruses, SV^d^: sendai virus, and OCT4 (O), SOX2 (S), KLF4 (K), c-MYC (M), NANOG (N), and LIN28 (L)^b^. Ion channels: *I*_Ks_: slow delayed rectifier K+ current; *I*_Kr_: rapid delayed rectifier K+ current; *I*_Na_: sodium channel current; *I*_K1_: inwardly rectifying K+ current; *I*_Ca,L_: L-type calcium current; and *I*_KAch_: acetylcholine activated potassium current.

**Table 4:** Human IPSC-derived patient-specific CPVT syndrome cell models.

Gene	Protein	Mutation	Donor cell types	Reprogramming method	References
*CASQ2*	Calsequestrin 2	p.D307H	Fibroblasts	LV^a^, OSKM^b^	[39]
*RyR2*	Ryanodine receptor 2	p.P2328S	Fibroblasts	RV^c^, OSKM	[47]
*RyR2*	Ryanodine receptor 2	p.F2483I	Fibroblasts	RV, OSKM	[45]
*RyR2*	Ryanodine receptor 2	p.S406L	Fibroblasts	RV, OSKM	[46]

LV^a^: lentiviruses, RV^c^: retroviruses, and OCT4 (O), SOX2 (S), KLF4 (K), and c-MYC (M)^b^.

**Table 5:** Human IPSC-derived patient-specific cardiomyopathy cell models.

Disease	Gene	Protein	Mutation	Donor cell types	Reprogramming method	References
Arrhythmogenic right ventricular dysplasia (ARVD)	*PKP2*	Plakophilin 2	c.2484C>T	Fibroblasts	РВ^a^, OSKM^b^	[49]
	*PKP2*	Plakophilin 2	c.2013delC	Fibroblasts	Epi^c^, OSKM	

Arrhythmogenic right ventricular dysplasia (ARVD)	*PKP2*	Plakophilin 2	c.972InsT/N	Fibroblasts	RV^d^, OSK^b^	[48]

Arrhythmogenic right ventricular dysplasia (ARVD)	*PKP2*	Plakophilin 2	c.1841T>C (p.L614P)	Fibroblasts	RV, OSKM	[54]

Dilated cardiomyopathy	*DES*	Desmin	с.940C>T (p.A285V)	Fibroblasts	RV, OSKM	[52]

Barth syndrome (dilated cardiomyopathy)	*TAZ1*	Tafazzin	c.590G>T, p. G197V	Fibroblasts	LV^f^, OSKM	[60]
			c.110-1AG>AC			
			170G>T, p. R57L			

Dilated cardiomyopathy	*TNNT2*	Troponin T type 2 (cardiac)	p.R173W	Fibroblasts	LV, OSKM	[51]

Hypertrophic cardiomyopathy	*MYH7*	Myosin heavy chain beta	p.R663H	Fibroblasts	LV, OSKM	[50]

LEOPAPD syndrome (hypertrophic cardiomyopathy)	*PTPN11 *	Protein tyrosine phosphatase, nonreceptor type 11	p.T468M	Fibroblasts	RV, OSKM	[62]

PB^a^: PiggyBac, RV^d^: retroviruses, LV^f^: lentiviruses, Epi^c^: episomes, and OCT4 (O), SOX2 (S), KLF4 (K), and c-MYC (M)^b^.

**Table 6:** Summary of published studies in cardiovascular disease with patient-specific iPSC.

Disease name	Cell type made	Phenotype displayed in iPSC-derived cells	Drug tested	References
Arrhythmogenic right ventricular cardiomyopathy/dysplasia	CMs	Reduced expression of plakophilin-2 and plakoglobin; evidence of myofibril disorganization; elevated lipid content relative to control CMs when they are exposed to adipogenic differentiation media	Nifedipine-inhibited contraction; isoproterenol increased contraction rate	[54]

Barth syndrome	CMs	Impaired cardiolipin biogenesis; ROS production was markedly increased and ATP levels were significantly lower; maximal electron transport chain activity was severely impaired in CMs	Linoleic acid improved sarcomere organization and increased twitch stress to nearly normal levels; mitoTEMPO treatment normalized sarcomere organization and contractility	[93]

Carnitine palmitoyltransferase II (CPT II) deficiency	Myocytes	CPT II-deficient myocytes accumulated more palmitoylcarnitine	Bezafibrate reduced the amount of palmitoylcarnitine	[94]

CPVT	CMs	Immature cardiomyocytes with less organized myofibrils and enlarged sarcoplasmic reticulum cisternae and reduced number of caveolae; DADs; oscillatory arrhythmic prepotentials; after-contractions and diastolic [Ca^2+^]_*i*_ rise	None	[39]

CPVT	CMs	Higher amplitudes and longer durations of spontaneous Ca^2+^ transients; Ca^2+^ release events after repolarization; abnormal Ca^2+^ response to phosphorylation induced by increased cAMP levels	None	[45]

CPVT	CMs	Elevated diastolic Ca^2+^ concentrations, a reduced sarcoplasmic reticulum Ca^2+^ content, and an increased susceptibility to arrhythmias	Dantrolene restored normal Ca^2+^ spark properties and rescued the arrhythmogenic phenotype	[46]

CPVT	CMs	Similar to above, but also evidence of early afterdepolarizations (EADs)	Flecainide and Thapsigargin blocked ads-beta blockers improved Ca^2+^ transient anomalies	[95]

CPVT	CMs	Aberrant Ca^2+^ cycling resulting in DAD and EAD	None	[47]

Familial dilated cardiomyopathy	CMs	Punctate sarcomeric *α*-actinin distribution; altered Ca^2+^ handling, decreased contractility	Norepinephrine markedly increased the number of CMs with punctate sarcomeric *α*-actinin distribution from DCM iPSC clones; metoprolol improved myofilament organization and significantly prevented aggravation of the DCM iPSC-CMs that is induced by norepinephrine treatment	[51]

LEOPARD syndrome	CMs	CMs are larger and have a higher degree of sarcomeric organization and preferential localization of NFATC4 in the nucleus compared to normal CMs	None	[62]

LQT1	CMs	Longer and slower repolarization velocity; abnormal subcellular distribution of R190Q KCNQ1; reduction of outward K^+^ current	Isoproterenol induced EAD was prevented by propranolol, simulating clinical LQT1	[24]

LQT2	CMs	Prolongation of the action potential duration; reduction of potassium current *I*_Kr_; EADs	Nifedipine: complete elimination of EADs; pinacidil: abolished EADs; ranolazine: pronounced anti-EAD effect at both cellular and multicellular level	[26]

LQT2	CMs	Same as above	Nicorandil and PD118057: action potential shortening and reduction of EADs; E4031: induced EADs; isoprenaline induced EADs and was blocked by nadolol and propranolol, simulating clinical treatment	[27]

LQT3	CMs	Dysfunction in Na^+^ channel gating, increase in *I*_NaL_, right-shifted steady-state channel availability, and faster recovery from inactivation	Mexiletine corrects Na^+^ channel inactivation	[30]

LQT3	CMs	Significantly prolonged APD and in patient-derived V-like hiPSC-CMs during the spontaneous contraction and during electrical pacing. Tetrodotoxin-sensitive late Na^+^current (dA/dF) was significantly larger in patient-derived hiPSC-CMs	Mexiletine reduced the late Na^+^ current, moderate effect of mexiletine in shortening the APD	[31]

LQT8 (Timothy syndrome)	CMs	Irregular contractions; excessive Ca^2+^ influx; prolonged action potentials; irregular electric activity; abnormal Ca^2+^ transients	Roscovitine normalized the Ca^2+^ defects and improved channel inactivation	[5]

Overlap syndrome of cardiac sodium channel disease	CMs	Significant decrease in *I*_Na_ density and upstroke velocity; a larger persistent *I*_Na_ leading to an increased persistent *I*_Na_	None	[33]

Marfan type 1	Mesenchymal cells	Elevated TGF-*β* signaling; inhibited osteogenesis and spontaneous chondrogenesis	None	[96]

Pompe disease (infantile onset)	CMs	Glycogen accumulation; ultrastructurally abnormal mitochondria; accumulation of autophagosomes; carnitine deficiency	L-carnitine increased O_2_ consumption and suppressed mitochondrial structural phenotype; treatment with rhGAA with autophagy inhibitor 3-MA normalized glycogen content	[97]

SAS	SMCs	Significantly lower level of ELN protein in SMCs and proliferate at a higher rate and migrate significantly faster in response to the chemotactic cytokine platelet-derived growth factor	Recombinant elastin or small GTPase RhoA rescues defective SM *α*-actin filament bundles	[69]
